# Acceleration of Anxiety, Depression, and Suicide: Secondary Effects of Economic Disruption Related to COVID-19

**DOI:** 10.3389/fpsyt.2020.592467

**Published:** 2020-12-15

**Authors:** M. Harvey Brenner, Dinesh Bhugra

**Affiliations:** ^1^Johns Hopkins University Bloomberg School of Public Health, Health Policy and Management, Baltimore, MD, United States; ^2^University of North Texas Health Science Center, Fort Worth, TX, United States; ^3^Medizinische Hochschule Hannover, Hanover, Germany; ^4^King's College London, London, United Kingdom

**Keywords:** COVID-19, economy, suicide, depression, national income loss, unemployment, recession, Great Recession

## Abstract

The SARS-CoV-2 (COVID-19) pandemic has contributed to increasing levels of anxiety, depression and other symptoms of stress around the globe. Reasons for this increase are understandable in the context of individual level factors such as self-isolation, lockdown, grief, survivor guilt, and other factors but also broader social and economic factors such as unemployment, insecure employment and resulting poverty, especially as the impacts of 2008 recession are still being felt in many countries further accompanied by social isolation. For those who are actively employed a fear of job and income loss and those who have actually become ill and recovered or those who have lost family and friends to illness, it is not surprising that they are stressed and feeling the psychological impact. Furthermore, multiple uncertainties contribute to this sense of anxiety. These fears and losses are major immediate stresses and undoubtedly can have long-term implications on mental health. Economic uncertainty combined with a sense of feeling trapped and resulting lack of control can contribute to helplessness and hopelessness where people may see suicide as a way out. Taking a macro view, we present a statistical model of the impact of unemployment, and national income declines, on suicide, separately for males and females over the life cycle in developed countries. This impact may reflect a potent combination of social changes and economic factors resulting in anomie. The governments and policymakers have a moral and ethical obligation to ensure the physical health and well-being of their populations. While setting in place preventive measures to avoid infections and then subsequent mortality, the focus on economic and social recovery is crucial. A global pandemic requires a global response with a clear inter-linked strategy for health as well as economic solutions. The models we have constructed represent predictions of suicide rates among the 38 highly industrialized OECD countries over a period of 18 years (2000–2017). Unemployment has a major effect on increasing suicide, especially in middle-aged groups. However, the impact of economic decline through losses of national income (GDP per capita) are substantially greater than those of unemployment and influence suicide throughout the life course, especially at the oldest ages.

## Introduction

The current epidemic of SARS-CoV-2 (COVID-19) has altered the way populations deal with stressors and resulting worries. The pandemic has affected directly or indirectly every individual on the planet but with varying individual and country responses. The impact of the pandemic on health is crucial but it also affects economic, educational and political aspects of life globally. Not surprisingly, the pandemic has led to a massive number of publications and research observations from cross-sectional to observational data on people in quarantine, self-isolation, shielding and others. A considerable and increasing number of professional society warnings and academic papers strongly suggest that the COVID-19 pandemic has resulted in anxiety- and depression-related illness, and potentially, suicide (1–7). These suggestions and assertions reflect both a fear of COVID-19 and resulting mortality as well as lockdowns and social distancing (this is a regrettable misnomer as the point is about physical distancing but socially we need ever more than before to be closer) intended to reduce infectious contact. Physical distance is needed to stop the infection but we do need to be socially closer to each other to support each other so that vulnerable people do not feel alone and isolated. Unfortunately, the term social distancing has taken off creating almost an egotistical validation of staying away from each other, thus we are using the term physical distancing (8). Those measures to reduce contact are well-recognized to cause damage to routine social relationships, internal family contacts and severely reduced interaction with the elderly and groups beset with compromised health due to chronic disease. But the intention of this article is to examine an entirely different aspect of the COVID-19 pandemic—namely, the indirect mental health effects of the major national and international recessions that have resulted from the attempt to contain COVID-19. Most obviously these recessions have involved an increase in United States unemployment of ~30 million jobless workers (collecting unemployment benefits) (9) and on an international level the result of potentially 400 million unemployed (10) and, at least equally important, declines in global economic output by $8.5 trillion over the next 2 years (11). The importance of the international recession and its implications for anxiety, depression, and suicide has as of yet not been clearly addressed in either the journalistic or scientific literatures. The impact of the previous recession from 2008 is still being felt in very many countries. This article specifically concentrates on the corollary, indirect mental health effects, especially suicide, resulting from the international recession, quite apart from the “direct” effects of COVID-19 on anxiety and depressive results. It is important to recognize that for many psychiatric disorders, symptoms themselves can be identified and used erroneously as diagnosis. For example, symptoms of feeling anxious or feeling low cannot and should not be seen as clinical anxiety or depression. However, a fair amount of research on Covid-19 has presented self-reported symptoms as clinical diagnosis.

In this paper, our focus is mainly on the mental health effects of this corollary to COVID-19 based strictly on damage to the economy. However, it is still too early to ascertain either what the intensity and duration of COVID-19 will ultimately be, or how it will affect national economies (12). In this paper we attempt to point out the types of factors that will be necessary to take into account when public health, and specifically mental health, service planners, in their attempt to create scenarios of optimal management of the mental health consequences of COVID-19 should consider. It will not be a simple matter to separate the direct mental health consequences of COVID-19, from the indirect, corollary effects of COVID-19 as it has brought considerable unemployment and income decline now and in the near and long-term future to national populations. For this reason, we have looked at a period of time, close to the COVID-19 era, namely the Great Recession of 2007–2010, and its aftermath, to examine how, “in an extraordinary recession,” economic decline in and of itself (i.e., absent major infectious epidemic) ordinarily produces extreme effects of anxiety and depression which have been associated with suicide. It is well-recognized that a recessional economy is related to heighted suicide for both sexes across the entire age spectrum, from at least age 15 to the end of the recorded life cycle. Most powerful are the effects of income loss which influence suicide at all ages, especially over 70, in contrast to the observable effects of unemployment which apparently influence increased suicide from age 15 throughout the life span apart from the most elderly populations of both sexes. These findings pertain to industrialized countries of the Organization for Economic Cooperation and Development (OECD), though it may well be that the observable effects are even more intense for low-income and middle-income emerging economies where the effect of the COVID-19 epidemic and corollary economic damage and mortality may be even more pronounced. Thus, the findings of this study on implications of economic loss to suicide serve as a hypothetical example as to what might happen in a worst case scenario for industrialized countries in trying to understand the corollary implications of COVID-19 to national economies, where suicide is the sentinel mental health outcome (13). Evidence is beginning to emerge that rates of psychiatric disorders such as anxiety and depression are rising in younger generations partly because of inter-generational inequalities where younger people feel that they are being left behind on a number of material parameters in addition to other factors such as urbanization and industrialization. We must acknowledge that this is a fast moving field of research and observations as well as interpretations are changing rapidly.

There has, rightly, been considerable epidemiological and media attention to the highly differential effects of COVID-19 as well as its economic implications on ethnic minorities and low socioeconomic populations in the industrialized world (14, 15). It is clear, from the epidemiology, as from economic analyses, that the populations most directly and severely affected by both the infectious disease and the corollary economic recession and its income/employment implications are “communities of color” and workers of relatively low income and education. In thinking about how efficiently and effectively public health and economic administrators should advance policy to minimize damage to mental health, it is clear that the immediate focus should be on these vulnerable populations. As in virtually all mental health and economic problems influencing national populations, epidemiology and economic analysis teach us the same lesson. It is that in virtually all major causes of illness and mortality, the health of lower socioeconomic groups, and especially lower socioeconomic ethnic minorities, the problems are most immediate and severe. This is partially a reflection of the sustained, and indeed increasing economic inequality that has been much the source of health disparities in industrialized countries (16).

### Study Aims

The principal aim of this paper is to identify the secondary effects of economic disruption in relationship to COVID-19 as these effects accelerate anxiety, depression and, especially, suicide. A statistical model of the impact of unemployment and national income declines on suicide, separately for males and females over the life cycle in developed countries during 2000–2017 will be central to the pinpointing of secondary effects of abrupt national recession. Suicide, in this case, is taken as a classic empirical indicator of anxiety, depression and anomie. This model thus provides the basis for estimating the potential simultaneous and lagged impact of unemployment and economic decline on suicide that accompanies the economic recession (and continues to be) intensified by COVID-19. Such a model allows us to anticipate the “purely” economic effects of COVID-19 on suicide, without considering the direct mental or physical health consequences of the COVID-19 infection. This permits consideration of the separate effects of economic recession that are amenable to policy mitigation through e.g., income support of the unemployed (especially long-term or permanent), small and large businesses that are damaged or terminated, and nationally financed investment in healthcare and educational personnel and social welfare. This analysis provides a structural basis for economic and health policy makers to take into account the mental health consequences of their prospective decisions. At the same time, the statistical models provide a basis for understanding the economic and political foundations of national-level suicide rates and their relations to official mental health-based diagnoses of elevated mortality, anxiety and depression. An additional aim is to provide an overview of the epidemiological history of national economic and unemployment risk factors to suicide. Finally, we offer suggestions as to psychiatrically-oriented policies that could be used to mitigate the current mental health effects related to the economic accompaniments of COVID-19 in The Way Forward.

## Materials and Methods

The general theme garnered among reporters and some writing in professional journals is that there are two clear implications for harm to mental health resulting specifically from the COVID-19 epidemic. The first is that people are simply “fearful” of going to work and appearing in public gatherings for fear of infection and mortality—especially among those with chronic cardiovascular, diabetic or asthmatic conditions as well as persons over 65. Additionally, there is the active and palpable fear of job and income loss, not only by those who are concerned about returning to normal social life, but those who have actually become ill or lost family and friends to illness and mortality in the COVID-19 pandemic as well as those who had insecure jobs in the first place. There is beginning to be some subjective reportorial literature pertaining to grief, without reference to the very extensive academic literature on stress, but with tacit or implicit knowledge that such fears and losses pertaining to health are major immediate stresses and can have long-term implications on mental health, analogous to those of what is now understood to be a classic PTSD series of events. In days of lockdown, with the loss of family members and inability to attend their funerals or even say goodbye itself can be seen as traumatic events.

But how can we reasonably predict—even in scenario terms—what the effects of COVID-19, and its corollary mental health disturbances would really look like? It is necessary to get answers to this in order to plan for public health, medical, economic, and specifically mental health policies.

### Lack of Statistical Data on Mental Health Outcomes

However, in none of these suggestive articles are there any statistical analyses (4–6, 17, 18). These very effusive and “common sense” observations by the press and mental health professionals seem to be considered so obvious as to not require further substantiation through statistical data, despite the fact that elaborate attempts have been developed by major universities, the Centre for Disease Control (CDC), and the World Health Organization (WHO) to provide statistical background and forecasting in the development of the COVID-19 epidemic itself. Part of the reason that statistical data on mental health implications on COVID-19 have not been forthcoming is that the typical sources of such data, including epidemiological studies and nationally recorded suicide rates, have required from 1 to 3 years before such data are actually gathered, and sufficiently refined and validated in order to be peer reviewed or located in national databases. As a result, despite considerable sentiment in the press and in initial suggestions in scientific literature of assertions of major mental health effects, the data have not been available to substantiate the prevalence, or intensity or the lethality of such mental health effects. These types of mental health effects have often been found in emergency calls to requests for urgent mental health counseling, interpersonal violence, threat to personal and friendship relationships, accidents, heavy use of alcohol and drugs as well as suicide attempts.

### Research Approaches in Recession and Suicide

The principal intellectual challenge for this paper is to produce evidence that will allow us to develop scenarios as to how the most current recession, based on shutdown of national economies in relation to COVID-19, will ultimately influence population mental health. Once again, it has been seen as “obvious” in journalistic accounts that the main mental health effect would arise out of *fear* of the COVID-19 infection itself and related mortality, as well as of potential losses of employment and income associated with that infection. Entirely missing thus far, however, has been data that would deal with a recent period of time where recessional economic losses have quantitatively influenced mental health without the influence of a major infectious pandemic. We would thus need to develop an empirical basis for understanding how, in the COVID-19 pandemic era, a significant proportion of measures of disturbed mental health would be influenced by deterioration of the economy—apart from what the COVID-19 implications of fear of infection and mortality would separately have on mental health.

This is not such an unusual problem, but rather one that has been more recently discovered as a potential epidemiological quagmire. We find this problem of separation of effects in virtually all major disaster research, where the primary research impulse is to identify the earliest short term influence of the disaster (e.g., floods, hurricanes, earthquakes, etc.) on those who immediately experience the disasters in terms of their on-the-spot threat to their lives and health. However, the losses of homes, occupations, family relations, and the elements of civilization surrounding the disaster have often been assessed in terms of their implication for mental health but not with COVID−19. This is the case, although it has been clear from the beginnings of research on life events, that these secondary, or corollary, phenomena influencing the direct economic and social relations consequences of the disaster, could have at least equal impact on the longer-term health situations (19). These corollary effects greatly concern persons indirectly subject to disasters and those in the larger surrounding communities which also feel the subsequent effects of those disasters, though not the immediacy of the natural events.

### Problem of Suicide Definition

In this paper we use suicide as a mental health outcome that would provide a sense of how mental health would be influenced by the economic implications of the COVID-19 recession. Because the inherent problems of measurement in suicide epidemiology are so complex, researchers have generally shied away from trying to “control” for the complicating effects of the measurements themselves. We acknowledge that there are clear problems in definitions and measuring rates of suicide. In the present research, we try to adjust, wherever possible, for some of the more important issues in the measurement of suicide. The first problem, given the available data is the issue of definition. When national figures on suicide rates are given in official records, can we assume that such suicide rate measures give us a reasonably accurate estimate of the volume of true suicides in the population at any historical point? It is well-known that many sources of mortality recorded, such as unintentional accidents, poisonings, drownings, mortality due to alcohol, and substance abuse, may all contain considerable elements of suicidal intent. How shall the medical examiner determine in a given case, for example, whether the single car accident embodied suicidal intent, or for the other categories of accidental or unintentional deaths mentioned above? There is, of course, the major national or legal element of the designation of a death as a suicide, in that the society is concerned that the reputation of the person identified as a suicide will be greatly harmed through stigma or long-term psychological damage to friends, parents, and offspring. In many countries, suicide remains an illegal act so families will do everything in their power to present the act as an accident. Furthermore, it is difficult to know whether the suicide is a result of infection (as a few cases have been reported in the media) or a result of economic pressures. Add to this the common assumption among epidemiologists that all deaths are the subject of multicausal factors, and comorbidities, and under the best of circumstances determination of an overall causal risk of mortality due to suicide is fraught with intellectual and societal problems.

Further, exacerbating this issue of definition lies in trying to determine time sequences. Thus, the heightened risk of suicide due to alcohol abuse may result in mortality that is not easily attributable to suicide, but the reaction of a loved one to such mortality could eventuate in suicide. Such reactions are not uncommon in the case of widow or widower suicides upon learning of the deaths of a spouse, for example (20–22). And a similar literature has been reported for adolescents (23). It is frequently difficult to ascertain whether illness or disturbed life circumstances due to alcohol or drug abuse or accidents or other trauma, themselves embody suicidal intent—or result in reactions of persons closely related who then go on to actually suicide.

The ultimate definitional question then is to what degree do deaths attributable to factors such as accidental poisoning, traffic accidents, or drug abuse represent suicides and need to be added to the category of suicides, perhaps in a broader concept such as “deaths of despair.” Another complicating factors is what Durkheim called anomie (in this case caused by the pandemic) and deserves further detailed study.

### Estimation Methods Related to Cultural Distinctions

An additional estimation problem arises when, as in the current analysis, we deliberately include different countries, so as to try to bring about a general understanding of the factors influencing suicide as a human problem, rather than one confined to suicide in a given country. These issues may be distinct from those of definition or other cultural or legal determinants which make suicide more or less likely in a specific society. The key problems here are cultural determinants of suicide including such elements as honor, bravery, social integration, individualism, meritocracy, and—perhaps especially—religion and its taboos with respect to taking one's own life. A related issue of cultural determination arises in discussion of gender differences in suicide. Here, considerations are generally given to masculinity and/or its assumed attributes of risk taking, bravery or dominance.

### Multivariable Estimation

Combined with economic factors and a sense of feeling entrapped and consequently a lack of control can contribute to helplessness and hopelessness seeing suicide as a way out thereby combining the impact of social changes due to anomie and economic changes as a result of the pandemic.

As yet, no statistical evidence exists for the very recent effect in the COVID-19 era of the radical increase in unemployment (e.g., 20% unemployment)—without the direct effects of COVID-19. There have been extensive qualitative discussions of the impact of unemployment on mental health outcomes in journalistic reports and academic papers very recently. These accounts have made reference to anecdotal data through interviews and literature reviews, and typically have made reference to how this might be playing out in real time, considering the great magnitude of such potential effects given a potential 15–20% unemployment rate. Several of these references made inferences from the most recent dramatic increase in unemployment occurring in the Great Recession of 2007–2009, when no major infectious disease epidemic occurred. Rather, the period just prior during, and following, the Great Recession was one which coincided with several mental disorder-related trends in industrialized societies. These include epidemic-like movements in alcohol consumption and abuse, drug abuse, drug poisonings, unintentional accidents, divorce rates, and other indications of family instability such as inter-personal gender based violence, child abuse etc. On the other hand it is recognized that high body mass index often attributed to behavioral factors can make people more vulnerable to COVID-19 related deaths (24, 25). BMI trends worldwide, but especially in industrialized countries have, in recent literature, been increasingly associated with disturbances to mental health, and, potentially, to increases in clinical depression (26) and also with increased likelihood of mortality due to COVID-19. These journalistic and scientific reports often melded, perhaps unintentionally, recent reports of psychological distress with the outcome of such trends, bearing in mind the long-term economic damage of the Great Recession. But, most recently, the damaged mental health assumptions emanating from this type of literature has found its way into the accurate reports of COVID-19 mental distress, focusing particularly on fear and loss related to infection, potential infection and actual COVID-19 mortality. This has given us a rather mixed picture of the blending of mental health trends of various origins with the anxiety and depression assumed to arise from the COVID-19 pandemic.

Thus, multifactorial origins of several damaging mental health trends, from the potential mental health effects of COVID-19 itself start to emerge. However, in the current COVID-19 era these journalistic reports and scientific papers have almost uniformly failed to recognize the major distinction between the mental health sequelae of COVID-19 as an infectious disease process from the accompanying massive recessional effects brought about by efforts to contain the pandemic. Yet, familiarity with the mental health effects of national economic disturbances should very quickly have focused researchers' attention on the potential and great magnitude of anxiety and depression implications of massive national unemployment rates and losses of income and wealth arising out of losses in GDP per capita, wages, and social welfare outlays. It is entirely possible that countries often have clear economic plans and strategy but do not have a mental health plan or policy as Bhugra et al. in a survey of Commonwealth countries reported that less than half of the members had a mental health policy (27).

### Effects of Recession Without COVID-19 Impact

How can we develop an estimate of the potential separate and indirect effects of COVID-19 recessional losses on mental health—being a corollary effect of COVID-19—from the direct effects of the pandemic itself on compromised mental health? The easiest way to accomplish this is to examine the most recent period of large-scale employment and income losses on mental health, in the absence of the COVID-19 pandemic. That reference would be to the Great Recession and its aftermath, with large-scale economic damage, but over (what is now in the COVID-19 era) a shorter period.

In having a numerical estimate of the effects of the Great Recession on mental health outcomes, it would be possible to make a comparison to that of national economic disturbance during varying periods (i.e., lengths of time) of the COVID-19 pandemic. The challenge then would be to estimate, for example, the numerical implications of an increase in unemployment during the Great Recession with a similar actual numerical increase in unemployment during the COVID-19 era.

### Estimating Major Effect of GDP per Capita

In developing such estimates of the actual vs. the potential impact of national recession on mental health outcomes, it is, in our view, of great importance to additionally separate the effects of short- and long-term losses of employment from the effects of income loss. The reason is that even in very major recessions only a minority of the population suffers employment loss, while actually a substantial majority of the population suffers losses of income and wealth. To put the income issue in broader perspective, it should simply be pointed out that virtually any goal of individual persons in their ordinary life adaptation and behavior, from food security, poverty minimization, and obtaining the worthwhile things in life in a market economy, requires finances. At the national level, income per capita also involves government revenues which are essential to the provision of health care, education, scientific and technological investments—often in the biomedical sphere. And for the younger population, the income base is essential for career development, social mobility and family formation that are fundamental goals. Interestingly, there have been very few studies that have separately examined the effects of income loss in the short term, and especially in the long term given government policies of austerity, on mental health implications apart from employment losses. An intermediate literature, lying between company losses and employment losses has in the last several years been concerned with firm downsizing—especially in the wake of the Great Recession (28). This managerial approach to occupational mental health has almost uniformly been able to demonstrate damage to mental health outcomes, for small and large businesses. A more remarkable finding in this literature is that in the downsizing process even workers who remain employed appear to show increased disturbances to mental health (29).

The absence of attention to GDP declines in previous literature on the effects of recession on mental health is particularly problematic, since welfare payments, including unemployment payments and assistance to firms in maintenance of jobs, as well as health care access and expenditures, have suffered considerably with the decline in government revenues which have been the basis of austerity budgets. These have been largely evident in Europe but have also been documented in North America (30, 31).

### Control for Education and Other Confounders

Added to the most visible outcomes of government austerity on poverty minimization are declines in government investment in education. This has not only had very serious effects on the ability of younger workers to develop careers in times of recession, but have led to longer-term effects on life time earnings and loss of productivity gains. The latter point of productivity growth diminution is estimated to have important implications for at least the next generation of workers and governments (32, 33).

Further compounding previous analyses of mental health effects due to recession has been the lack of use of multivariable models predicting, e.g., suicide, but taking into account other major sources of risks than the immediacy of recession, such as alcohol and drug abuse or accidental mortality. And, as indicated earlier, the lack of control for such potential risks often hide significantly the inherent suicidal intent (or actuality), of suicidal behavior represented by such risks. Thus, the absence of control for such factors, at the very least, increases the risk of misestimation of the actual level of suicide that is contingent upon economic damage.

A perennial problem in the analysis of suicide, since at least the time of Durkheim, has been the population samples on the basis of which suicide is estimated or predicted (34). On the one hand, one would prefer, on statistical grounds, to have as large and representative a multicultural population as possible. In this way, it becomes easier to make general statements about the effects of particular risk factors, such as unemployment, on the broad nature of mental health. On the other hand, since it is widely acknowledged that cultural factors are of distinctive value in developing models to predict suicide, it is of special importance to focus one's statistical analysis on culturally homogenous societies where, cultural norms, values and beliefs can be controlled more easily. Fortunately, more recent approaches in epidemiology, often referred to as “econometric” allow the analyst to control for such cultural factors in a multisocietal framework by using “dummy” or binary variables to identify geographic or politically identified ecological areas (i.e., specific geographical areas = 1 other areas = 0). All in all, it is now feasible to construct models predicting mental health outcomes, which can include not only major national economic events, but control, within the same model for risk and definitional factors as well as regional distinctions which discriminate cultural and political attributes of regions (35).

### Country Differences and Opportunity Costs of Policy

Nevertheless, the choice of overall region to be the subject of statistical analysis remains fundamentally important. This is particularly true in the case of psychiatric outcomes, where, among the world's societies, there are large distinctions as to the psychiatric reliability of a suicidal diagnosis. In this paper, we therefore focus on uniform data available from the OECD, which is largely based on data from the most highly industrialized societies. The presumption is that data from these societies on suicide are likely to respect the psychiatric and scientific conventions of mental health diagnoses, and less likely to be heavily influenced by religious or other societal stigmas that would serve to contaminate criteria for coroners or medical examiners (official reports) (36).

### Welfare, Unemployment Benefits, Aid to Businesses

In modeling the prediction of suicide in industrialized societies, it is clearly important to take into account the variety of beneficial factors that might influence societal anxiety and depression. Important in this regard are professional social welfare efforts usually through government expenditure, to manage societal mental health problems. This is especially true since the relation between lower socioeconomic status and poor mental health is so widely acknowledged it is of special importance to concentrate on issues of poverty, homelessness, unemployment or insecure employment and long-term psychiatric disability in minimizing suicide. Thus, societies are faced with the usual issues of opportunity cost as they face political decisions involving physical and mental health. To what degree does the society concentrate on basic support of material living conditions and education as distinguished from more highly medicalized attempts to improve overall health or mental health levels. This involves intense political discussions which are important here but are not the direct subject of this paper. Nevertheless, in the statistical modeling process, one needs to bear in mind not only the level of society's overall income and wealth, but rather the specific monies allocated to promote differential societal goals that also promote health. This is clearly a limiting factor in the use of the GDP per capita as a primary source of influence on mental health; yet, with a reduced GDP per capita there is less governments can achieve, regardless of their competing policies. It is clear, then, that the sheer magnitude of GDP per capita is a prime limiting factor in how much governments can accomplish in order to improve mental health.

### Potential Effects of Mental Health Services

It is worth looking at the impact of COVID-19 on mental health in stages. For example, first stage of quarantine, self-isolation may bring with it certain stressors especially if individuals are living by themselves or nuclear family settings in many high income countries. Second stage will be of infection and isolation either at home or in hospital. Bereavement as a result of death of a loved one and inability to attend funerals in lockdown situations will affect coping with grief and may well-lead to abnormal grief reactions. In many countries an inability to perform rituals after a death can further add to distress and resulting depressive feelings. Some individuals may go on to experience survivor guilt. Each of these observable stages will affect mental health and well-being of individuals. In low income countries which may be socio-centric, additional pressures may play a role. Thus, the fear of catching the infection can lead to avoidance anxiety, the sense of being entrapped can lead to depression and grief reaction due to loss and bereavement followed by managing survivor guilt and each of these conditions can contribute to increased likelihood of self-harm or suicide. In all the preparations for dealing with the pandemic, the emphasis initially was on prevention and then treatment, the focus on mental health emerged later. In dealing with mental ill-health the focus must be on individual, family, community, and then national and global responses.

### Statistical Analysis

#### Pooled Cross-Sectional Time Series (PCSTS) Analysis

It is common that for some observational studies, observations are available over a sequence of points in time, e.g., countries and years as in our case. Taking into account only one dimension, i.e., space or time, would restrict us to perform classical cross-sectional or time series regression analysis. Using more advanced techniques (37, 38), i.e., pooled cross-sectional time series analysis, allows us to model simultaneously the space and time dimension. The usefulness of the PCSTS approach for health care systems analysis is described e.g., in Reibling (39).

The PCSTS method combines two approaches. The more familiar is cross-sectional analysis, where, in this case, countries of the OECD are the units of analysis (i.e., 38 countries). We examine multiple cross-sectional analyses corresponding to the 18-year period-−2000–2017 for which all of the data representing the individual variables are available for the OECD countries. All variables used in these PCSTS analyses are based on aggregated data—i.e., population rates rather than individual-level data (40)^1^. In addition to the cross-sectionality of this procedure, the technique simultaneously entails time-series analysis, involving variations over time in the individual predicted variables and the outcome variable, suicide (41).

PCSTS models can be regarded as extensions of a common linear regression model where for the pooled observations the error term is split up in a unit specific term and a stochastic remainder disturbance. Different assumptions about the stochastic properties of the unit specific term raise two main PCSTS models: the fixed effects and the random effects estimator. The fixed effects model assumes that the unit specific term is non-stochastic and constant over time. The random effects model treats the unit specific term as a stochastic entity. Methodological details for both models and the estimation techniques are provided in Baltagi and Wooldridge. Disadvantages of the fixed effects estimator are that it cannot deal with variables that do not change over time and that it provides imprecise estimates when variables change only slowly over time (42). As our models include dummy variables which cover specific effects of selected countries, the random effects estimator is used for all models.

PCSTS is a well-established procedure in economics over the last 30 years and has recently been introduced in health service research and epidemiology (43, 44). The findings are easily replicable with enclosed data sources by a statistician using STATA.

#### Key Variables

Three types of variables included in this model are dependent, independent, controls.

##### Dependent

Age-adjusted suicide in international WHO database, according to coroner's and/or medical examiner's, ICD code separately by sex and age—for each OECD industrialized country.

##### Independent

Macroeconomic and unemployment variables. These are the basis of our hypothesis—as related to recession of 2008-10, depending on country.

##### Control

Divorce, etc.; unintended injuries (now the third highest cause of death in the United States 2020); accidents (especially automobile; single car); self-poisonings; drug overdoses; fire/burns; drownings.

These above control variables could actually represent suicides but for factors relating to stigma, or classification “error” given the specifications of the ICD code, these “causes” of death may in many instances be actual proximal mechanisms of suicidal death, where the mental “intent,” e.g., a state of depression, might be the true psychological state which underlay (i.e., were foundational to) these mechanisms of death (e.g., drug overdoses)—and may in fact represent suicidal behavior.

From a methodological point of view we want to hold constant other risks of suicide that could also be correlated with both economic changes and officially identified suicides. Without these controls, the effects of economic change could either be underestimated or overestimated.

### Forecasting of Effects of COVID-19 Recession on Mental Health and Suicide

The statistical models demonstrating the sheer implications of unemployment increases and national income (GDP per capita losses) over 2000–2017 provide the basis for understanding, and ultimately estimating, the potential future mental health and suicidal impact of the recessional phenomena during the ongoing, and rapidly continuing—in terms of its consequent production of economic recession. But at this point we do not know how intense or lengthy the COVID-19 recession will be among industrial democracies of the OECD. Equally important, we have no foreknowledge of what the individual governments may invest in unemployment, business, welfare, health care, and educational relief and stimulus to maintain economic stability and mitigate poverty as COVID-19 and its sequelae proceed. It is clear that different governments are responding in different ways. Further, the epidemiological literature indicates that the economic impact of employment and income loss and poverty may lag over a range of at least 5–10 years, if not a generation. Therefore, the coefficients showing twenty-first century relations between income and employment loss and mental health, must, in practical policy discussions, be stated in terms of scenarios that refer to potential policy decisions on the part of governments.

## Results

### The Suicide Models for Industrialized Countries

The models we have constructed represent predictions of suicide rates among the 38 highly industrialized OECD countries over a period of 18 years (2000–2017) (see Tables 1, 2). There are two sets of models, for males and females that separately demonstrate relations for 5-year age groups over the life course. All models contain at least three basic variables. These are the two economic variables representing the effect, firstly, of changes (fluctuations and trends) in GDP per capita, with a 5-year lag. The 5-year lag is intended to capture especially the effects of innovations in pharmaceuticals and medical procedures, generally effectuating lower mortality rates across the age spectrum. The GDP per capita is also the principal factor that is identified by economists to represent changes in the business cycle, where a decline in GDP over at least 2 quarters by definition represents recession. The other major economic variable, perhaps more famous journalistically for its representation of recession, is the unemployment rate as a proportion of the total labor force of workers over the age of 15.

**Table 1:** Prediction of suicide death rate (intentional self-harm, ICD-10 X60-X84) in male population.

**Predictor**	**Coef**.	***P*-value**	**95% CI lo**	**95% CI hi**
Five year lag of GDP per capita at PPP in '000 of 2011 international dollars	−0.176	0.000	−0.226	−0.126
Unemployment rate as % of total labor force age 15+	0.126	0.000	0.074	0.177
Mental and behavioral disorders due to psychoactive substance use death rate (ICD-10 F10-F19)	0.294	0.000	0.209	0.380
Road injuries death rate (ICD-10 V01-V89)	0.132	0.000	0.072	0.192
Exposure to fire, heat, and hot substances death rate (ICD-10 X00-X19)	0.883	0.000	0.664	1.102
Accidental poisoning by and exposure to noxious substances death rate (ICD-10 X40-X49)	2.064	0.000	1.522	2.605
Regional dummy (1 = Greece and Turkey, 0 = rest of the world)	−13.367	0.000	−18.369	−8.366
Regional dummy (1 = Central America, 0 = rest of the world)	−11.078	0.000	−16.083	−6.073
Regional dummy (1 = Eastern Asia, 0 = rest of the world)	14.605	0.000	9.601	19.609
Regional dummy (1 = Western Europe, 0 = rest of the world)	5.859	0.000	2.854	8.863
Regional dummy (1 = Slovenia and Hungary, 0 = rest of the world)	11.795	0.000	6.836	16.755
Constant	15.555	0.000	12.877	18.232

*Random-effects pooled GLS regression for 38 OECD countries and 18 years (2000–2017), strongly balanced data. Overall R-square 0.82. All death rates are age-adjusted per 100,000 male population*.

**Table 2:** Prediction of suicide death rate (intentional self-harm, ICD-10 X60-X84) in female population.

**Predictor**	**Coef**.	***P*-value**	**95% CI lo**	**95% CI hi**
5 year lag of GDP per capita at PPP in '000 of 2011 international dollars	−0.129	0.000	−0.143	−0.116
Unemployment rate as % of total labor force age 15+	0.031	0.003	0.010	0.051
Mental and behavioral disorders due to psychoactive substance use death rate (ICD-10 F10-F19)	0.434	0.000	0.355	0.513
Adverse effects of medical treatment death rate (ICD-10 T36-T50, T80-T88)	0.161	0.416	−0.228	0.551
Regional dummy (1 = Greece and Turkey, 0 = rest of the world)	−2.816	0.008	−4.897	−0.734
Regional dummy (1 = Central America, 0 = rest of the world)	−3.492	0.001	−5.624	−1.359
Regional dummy (1 = Eastern Asia, 0 = rest of the world)	9.396	0.000	7.304	11.487
Regional dummy (1 = Western Europe, 0 = rest of the world)	3.959	0.000	2.533	5.385
Regional dummy (1 = Australia and New Zealand, 0 = rest of the world)	2.302	0.030	0.225	4.378
Regional dummy (1 = Slovenia and Hungary, 0 = rest of the world)	2.967	0.005	0.884	5.049
Regional dummy (1 = Scandinavia, 0 = rest of the World)	3.871	0.000	2.308	5.434
Regional dummy (1 = Switzerland and Luxembourg, 0 = rest of the world)	3.324	0.006	0.973	5.676
Constant	6.157	0.000	5.237	7.077

*Random-effects pooled GLS regression for 38 OECD countries and 18 years (2000–2017), strongly balanced data. Overall R-square 0.72. All death rates are age-adjusted per 100,000 female population*.

The third variable common to all models (irrespective of gender and age groups) is the mortality rate for substance use disorders (i.e., mental and behavioral disorders due to psychoactive substance use death rates [ICD-10 F10-F19]). This ICD categorization of mortality typically represents addictive behavior or abuse of especially alcohol and illicit drugs being used for psychopharmacological reasons in mood alteration, generally not under the regulation and prescription of medical personnel. The literature is unable to fully discriminate between the effects of substance abuse that is the product of suicidal intent, from the effects of substance abuse that causally results in suicide (but which may not have been originally intended). We include the substance abuse disorder/death rate as a means of controlling for the fact that the nationally designated suicide rate may insufficiently refer to the substance abuse death rate that involves suicidal intent or consequences.

Additional variables predicting suicide among the industrialized countries are differentiated between males and females (see Table 5). Especially important for males is the ICD category poisonings death rate, which often results from inadvertent overdose of various poisonous substances (45), including opioids which often involve abuse of pharmaceutically prescribed drugs for pain and psychophysiological reasons, including opioids. Once again, it is not evident whether a large proportion of these drug poisonings imply suicidal intent, or whether a suicidal outcome may result from overdosing of these substances. It is not at all clear, theoretically, why male populations should be more subject to the poisoning death rate as it may relate to suicide data, but our initial observations have been that the male poisoning death rate is especially correlated with the overall male suicide rate, whereas the female poisonings death rate is not significantly associated with the female suicide rate. On the other hand, the diagnostic category “adverse effects of medical treatment” death rate of females is highly correlated with the female suicide death rate—especially for younger age groups—but these adverse effects of medical treatment are not significantly related to male suicide rates. It is not immediately evident how gender for these diagnostic categories of mortality should differentially affect male and female suicides. However, there are indications in the literature that females in industrialized countries with mental disorders, and especially with suicidal attempts, are more likely to receive medical/psychiatric treatment than males (46, 47). It is also possible that females, evidencing suicidal intent in their use of prescription drugs, may be more likely to be classified as suicides than males. Once again, it would require careful analysis to determine whether, and to what degree, the overuse of pharmaceutical drugs implies suicidal intent or, rather, that the pharmaceutical drug overuse results in suicide.

The number of potential suicide-related categories that significantly predict suicide are somewhat longer in the case of male populations as compared to females. In the case of males, the death rate category of “road traffic injuries” is significantly correlated with male suicide, as are, in addition, death rates associated with “fire, heat, and hot substances.” The literature, here again, is not very clear as to why these categories of death should be more closely associated with male suicide. However, there are studies of suicide patterns that generally indicate that males are considerably more likely to use violent means in suicidal acts, whereas female suicides are more typically associated with relatively passive methods, including substance abuse and drug overdose (48, 49).

### Life Cycle Distribution

**Figure 1:**
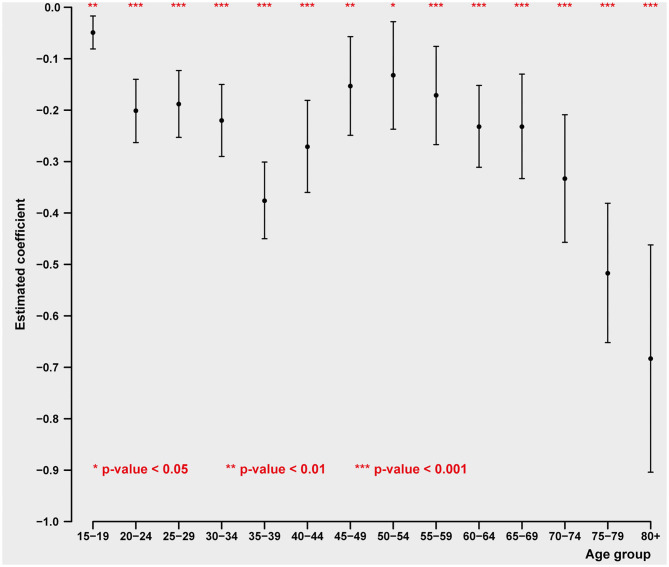
Impact of GDP pc on age-specific male intentional self-harm (ICD-10 X60-X84) death rate (pooled cross-sectional time series regression, 38 OECD states, years 2000–2017). Estimated coefficients with 95% confidence intervals. Adjustment variables: unemployment rate, mental, and behavioral disorders due to psychoactive substance use (ICD-10 F10-F19) death rate, road injuries (ICD-10 V01-V89) death rate, exposure to fire, heat, and hot substances (ICD-10 X00-X19) death rate, accidental poisoning by and exposure to noxious substances (ICD-10 X40-X49) death rate, regional dummies.

**Figure 2:**
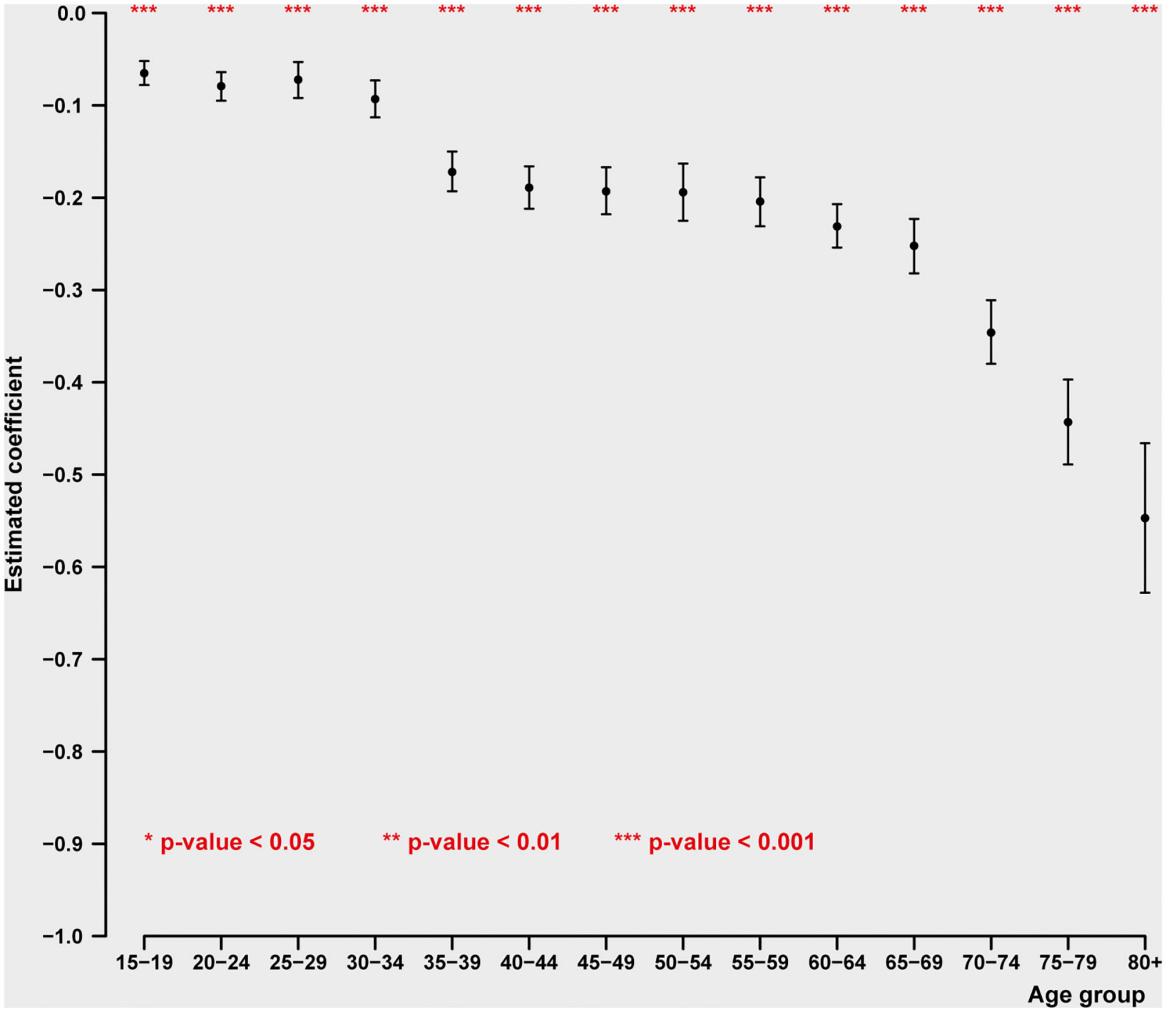
Impact of GDP pc on age-specific female intentional self-harm (ICD-10 X60-X84) death rate (pooled cross-sectional time series regression, 38 OECD states, years 2000–2017). Estimated coefficients with 95% confidence intervals. Adjustment variables: unemployment rate, mental, and behavioral disorders due to psychoactive substance use (ICD-10 F10-F19), adverse effects of medical treatment death rate (ICD-10 T36-T50, T80-T88), regional dummies.

Male and female suicides differ considerably in their pattern of relationship to the principal economic phenomena GDP per capita and unemployment (see Figures 1, 2 and Tables 3, 4). Males at every single one of 14 age groups 15–19 through 80+ show highly significant inverse relations to GDP per capita. As GDP per capita rises, male suicides inevitably decline. The first portion of the life cycle at which the inverse relationship between GDP per capita and suicide declines is between 20–24 and 40–44, with the peak of these inverse relationships at 35–39 and 40–44. The youngest period of life during which male suicides strongly decline in relation to economic growth is in early middle age, i.e., 30–44. In other words, economic damage caused by decline in national income and wealth has an especially powerful damaging effect on elevating male suicides in early middle age.

**Table 3:** Impact of key economic variables (5 year lag GDP pc, unemployment rate) and key mortality rates [substance use (ICD-10 F10-F19), road injuries (ICD-10 V01-V89), fire/heat (ICD-10 X00-X19), and poisoning (ICD-10 X40-X49)] on male intentional self-harm (ICD-10 X60-X84), total age adjusted mortality and 14 age groups.

	**5 year lag GDP**		**Unemp rate**		**Substance use**		**Road injuries**		**Fire, heat**		**Poisonings**	
	**Coeff**.	***P*-value**	**Coeff**.	***P*-value**	**Coeff**.	***P*-value**	**Coeff**.	***P*-value**	**Coeff**.	***P*-value**	**Coeff**.	***P*-value**
Total age adjusted	−0.176	0.000	0.126	0.000	0.294	0.000	0.132	0.000	0.883	0.000	2.064	0.000
15-19	−0.049	0.003	0.070	0.000	0.960	0.000	0.114	0.000	4.129	0.000	2.443	0.000
20-24	−0.201	0.000	0.093	0.000	0.370	0.000	0.137	0.000	3.389	0.000	2.990	0.000
25-29	−0.188	0.000	0.042	0.022	0.192	0.000	0.170	0.000	1.905	0.000	4.216	0.000
30-34	−0.220	0.000	0.103	0.015	0.178	0.000	0.209	0.000	1.620	0.000	1.845	0.000
35-39	−0.376	0.000	0.167	0.000	0.384	0.000	0.162	0.000	1.203	0.000	1.078	0.004
40-44	−0.271	0.000	0.352	0.000	0.474	0.000	0.324	0.000	0.328	0.012	1.990	0.000
45-49	−0.153	0.002	0.457	0.000	0.382	0.000	0.386	0.000	0.639	0.000	1.636	0.000
50-54	−0.132	0.013	0.479	0.000	0.303	0.000	0.206	0.001	0.211	0.067	3.340	0.000
55-59	−0.171	0.000	0.062	0.000	0.207	0.000	0.007	0.895	1.077	0.000	2.168	0.000
60-64	−0.232	0.000	0.235	0.000	0.090	0.007	0.029	0.445	0.749	0.000	1.380	0.000
65-69	−0.232	0.000	0.122	0.061	0.019	0.700	0.193	0.000	0.661	0.000	1.371	0.000
70-74	−0.333	0.000	0.000	0.998	−0.261	0.000	0.082	0.089	0.000	0.000	2.453	0.000
75-79	−0.517	0.000	0.027	0.718	−0.455	0.000	0.109	0.016	0.876	0.000	−2.943	0.000
80+	−0.683	0.000	0.093	0.460	−0.737	0.000	0.052	0.385	0.391	0.003	0.212	0.773

*Pooled cross-sectional time series regression, 38 OECD states, years 2000–2017. Estimated coefficients and P-values*.

**Table 4:** Impact of key economic variables (5 year lag GDP pc, unemployment rate) and key mortality rates [substance use (ICD-10 F10-F19), adverse effects of medical treatment (ICD-10 T36-T50, T80-T88)] on female intentional self-harm (ICD-10 X60-X84), total age adjusted mortality and 14 age groups.

**Total age adjusted**	**5 year lag GDP pc**		**Unemployment rate**		**Substance use**		**Adv effects med treatment**	
	**Coeff**.	***P*-value**	**Coeff**.	***P*-value**	**Coeff**.	***P*-value**	**Coeff**.	***P*-value**
	−0.129	0.000	0.031	0.003	0.434	0.000	0.161	0.416
15–19	−0.065	0.000	0.018	0.000	1.138	0.000	12.650	0.000
20–24	−0.079	0.000	0.021	0.000	0.431	0.000	12.288	0.000
25–29	−0.072	0.000	0.031	0.055	0.237	0.000	10.119	0.000
30–34	−0.093	0.000	0.019	0.251	0.216	0.000	5.026	0.000
35–39	−0.172	0.000	0.055	0.020	0.341	0.000	5.532	0.000
40–44	−0.189	0.000	0.085	0.000	0.403	0.000	3.268	0.000
45–49	−0.193	0.000	0.105	0.000	0.405	0.000	4.191	0.000
50–54	−0.194	0.000	0.088	0.000	0.448	0.000	3.689	0.000
55–59	−0.204	0.000	0.066	0.003	0.439	0.000	0.009	0.979
60–64	−0.231	0.000	0.050	0.014	0.408	0.000	0.198	0.335
65–69	−0.252	0.000	0.070	0.005	0.311	0.000	0.243	0.100
70–74	−0.346	0.000	0.069	0.015	0.357	0.001	−0.130	0.188
75–79	−0.443	0.000	0.050	0.181	0.697	0.000	−0.081	0.294
80+	−0.547	0.000	0.057	0.379	0.303	0.034	−0.028	0.535

*Pooled cross-sectional time series regression, 38 OECD states, years 2000–2017. Estimated coefficients and P-values*.

The second period in which male suicides are highly sensitive to economic changes is after the age of 60. That sensitivity is moderately strong between 60 and 74, but rises to a peak in the ages over 75 (75–79 and over 80). Once again, this means that the period of life for males during which declines in material well-being are most likely to be associated with increased suicide are over the age of 60, and especially over 75. The age of retirement varies across countries.

This is in contrast with the age specific pattern of suicide for women. In the case of females, we find, remarkably, what is virtually a linear, dose-response relationship between age and suicide. The older the age of the female population, the more likely is the occurrence of suicide in relation to declines in GDP per capita. Somewhat similar between the sexes is the unusually strong increases in suicide in the later stages of the life cycle, 75–79 and over 80 years of age. Thus, especially for women, losses of income appear increasingly important with increases in aging. And the most powerful effect of income loss in relation to female suicide is in the very late ages of life.

### Unemployment and Suicide Over the Life Cycle

**Figure 3:**
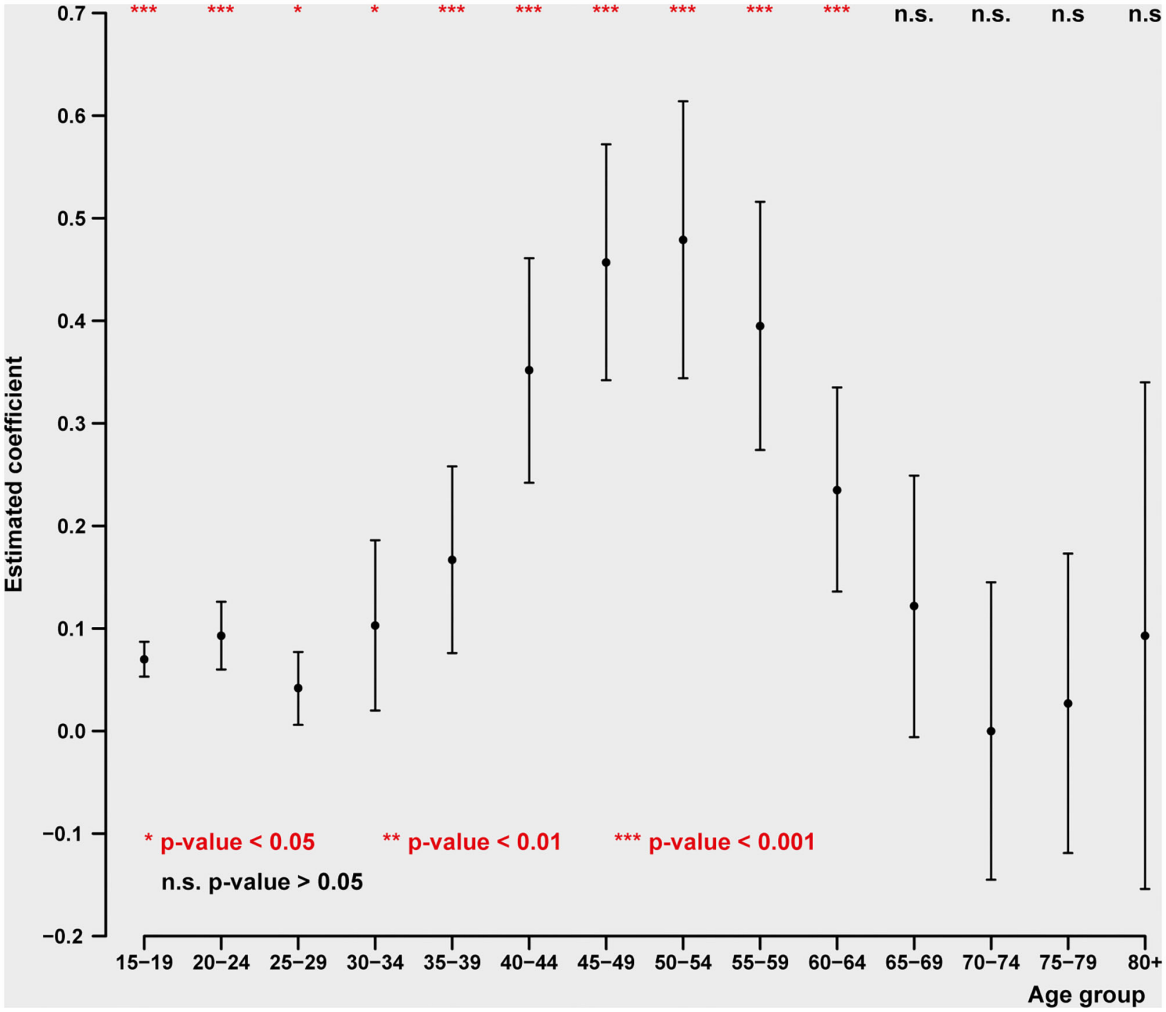
Impact of unemployment rate on age-specific male intentional self-harm (ICD-10 X60-X84) death rate (pooled cross-sectional time series regression, 38 OECD states, years 2000–2017). Estimated coefficients with 95% confidence intervals. Adjustment variables: GDP pc, mental, and behavioral disorders due to psychoactive substance use (ICD-10 F10-F19) death rate, road injuries (ICD-10 V01-V89) death rate, exposure to fire, heat, and hot substances (ICD-10 X00-X19) death rate, accidental poisoning by and exposure to noxious substances (ICD-10 X40-X49) death rate, regional dummies.

**Figure 4:**
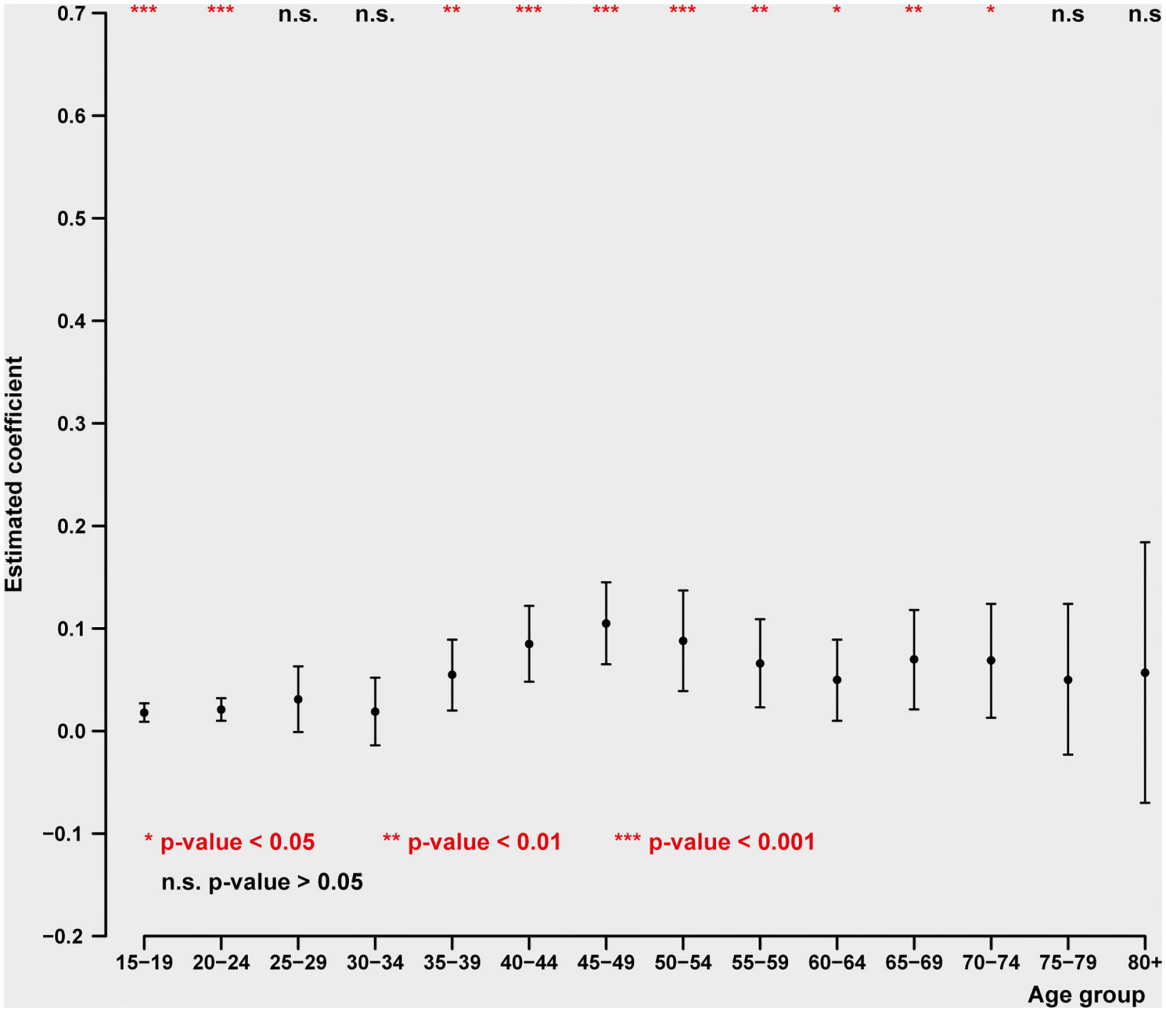
Impact of unemployment rate on age-specific female intentional self-harm (ICD-10 X60-X84) death rate (pooled cross-sectional time series regression, 38 OECD states, years 2000–2017). Estimated coefficients with 95% confidence intervals. Adjustment variables: GDP pc, mental, and behavioral disorders due to psychoactive substance use (ICD-10 F10-F19), adverse effects of medical treatment death rate (ICD-10 T36-T50, T80-T88), regional dummies. ICD-10 causes of death: (1) intentional self-harm (ICD-10 X60-X84); (2) mental and behavioral disorders due to psychoactive substance use (ICD-10 F10-F19); (3) road injuries (ICD-10 V01-V89); (4) exposure to fire, heat, and hot substances (ICD-10 X00-X19); (5) accidental poisoning by and exposure to noxious substances (ICD-10 X40-X49); (6) adverse effects of medical treatment (ICD-10 T36-T50, T80-T88).

In contrast to the relation of suicide to GDP change, for males the relationship between unemployment and suicide is highest in early and late middle age (40–64) and disappears entirely after the age of 70 (see Figure 3). This presumably reflects the duration of the usual working life and being laid off later in life, when there is little potential to find new employment. In the case of women, the relation between unemployment and suicide is generally weaker than that for men. The peak of the female relationship between ages 40–54 to unemployment nevertheless remains strong even in the ages 65–74 (see Figure 4), while, as in the case of males it disappears entirely after the age of 75.

### General Life Cycle Relations to “Deaths of Despair”

In principle, as shown in Tables 3, 4 the major effects of income loss should occur at two key stages in the life cycle. The first is at “middle age” of ~35–55 age groups; this represents both the height of job earnings as well as the period after which it is most difficult to find work following job loss.

The period over 65 encompasses the ages presumably least expected to be influenced by unemployment, since unemployment is lowest at this period. At the same time, the ages over 70 are those with the highest rates of poverty in industrialized countries (50). And we do find, for both sexes, at ages over 70, when suicide responds the most sensitively—i.e., in terms of the greatest increase—to declines in income per capita.

If we now examine the relations of potential mechanisms of suicide to nationally identified suicide rates, we find similar patterns. For male substance abuse and road injury (death rates), the relation of mortality to identified suicide (death rates) come to a broad peak level at 35–54 (see also Table 5). Fire, heat and hot substances, as well as poisonings, mortality are related to suicide mortality at somewhat later middle ages of ~55–69 and 40–74. Apart from this pattern for males, youthful mortality for substance abuse; fire, heat, and hot substances; and poisoning deaths also occur at another general peak at a range of 15–19 through age 35.

**Table 5:** Variables used to predict elevated suicide rates.

**Predictor variables**	**Data sources**	**Countr**.	**Time period**
Self-harm death rate per 100,000 of male age adjusted population	IHME Global Health Data Exchange	192	2000–2017
5 y/lag of GDP per capita, PPP (constant 2011 international $)	World Bank, International Comparison Program database	191	1990–2017
Unemployment rate (%) in total population 15+	ILOstat	189	1991–2017
Substance use disorders death rate per 100,000 of male age-standardized population	IHME. Global Health Data Exchange. GBD Results Tool	192	2000–2017
Road injuries death rate per 100,000 of male age adjusted population	IHME. Global Health Data Exchange. GBD Results Tool	192	2000–2017
Fire, heat, and hot substances death rate per 100,000 of male age adjusted population	IHME. Global Health Data Exchange. GBD Results Tool	192	2000–2017
Poisonings death rate per 100,000 of male age adjusted population	IHME. Global Health Data Exchange. GBD Results Tool	192	2000–2017
Self-harm death rate per 100,000 of female age adjusted population	IHME. Global Health Data Exchange. GBD Results Tool	192	2000–2017
Substance use disorders death rate per 100,000 of female age-standardized population	IHME. Global Health Data Exchange. GBD Results Tool	192	2000–2017
Adverse effects of medical treatment death rate per 100,000 of female age adjusted population	IHME. Global Health Data Exchange. GBD Results Tool	192	2000–2017

For women, a middle-age high level range in substance abuse mortality, correlated with female suicides, occurs at 35–39 through 60–64. But additionally high levels of substance abuse mortality related to suicide occur at the younger ages of under 19 through 30, and at the very high age range of 65–69 through 75–79. Perhaps most remarkable are the very strong correlations between female mortality identified with adverse effects of medical treatment and official female suicide rates. Unusually strong relations are found under age 30, with relatively strong relationships between the “standard” 30–54 (middle-aged) suicide rates, but no significant relations after age 54. There is a considerable likelihood that these strong relationships to suicide mortality are consequences of adverse relations to pharmaceuticals of which over 200 have been identified (51).

## Discussion

### Relation of Findings to the Literature

There is, certainly, an extensive and lengthy set of literature in psychiatry, psychology and the newer field of psychoneuroimmunology on stress as a source of mental health disturbances of many different varieties and, especially, of anxiety, depression, and suicide (52–54). This gives rise, on a scientifically professional level, to theoretical considerations that it is very likely that conditions such as COVID-19, and its direct ramifications for stress, will be sources of frank mental illness and suicide. But, surprisingly, this initial set of journalistic and professional suggestions for a stress basis of mental health disorders, resulting from COVID-19, heavily concentrate on fear of the infectious implications of COVID-19. The most surprising implication, however, is that very little attention appears to have concentrated on actual losses, emanating from the radical termination of jobs (especially in the industrialized world, and most especially in the United States), and even less attention seems to have been given to financial losses of income and wealth in the short and long term. This is rather surprising in view of the fact that much of the professional literatures on stress, life events, and economic losses, have concentrated for generations on both the mental and extensive “physical” effects—especially in illnesses that have been classically linked to acute and long-term stress, such as cardiovascular symptoms and mortality. Indeed, given the volume of chronic disease and accidental mortality, one might wonder whether the potential burden of illness and mortality resulting from economic losses might not be greater from those that are more directly affected than are assumed to follow from the fear of COVID-19 infection and mortality.

### Loss, Anxiety, and Depression

The scientific literature is now fairly extensive on the distinction between short and long-term effects of stress, especially as it would pertain to anxiety, depression and psychophysiologic changes. In particular, very short term stresses have been thought to actually be a source of beneficial stress—i.e., “eustress” as originally formulated by Selye (55). In the acute stress situation, under the assumption that it is indeed short term and will pass, the elevation of physiologic responses would tend to increase the likelihood that the subject can cope successfully, or deflect the stress itself so that its duration remains limited. On the other hand, longer-term, or chronic stress, including “daily hassles” (56) are generally thought to be sources of considerable mental health damage, and extensive harm to physiological function, through the emergence and sustained pattern of chronic disease (57). It is this depiction of long-term stress that is often associated with declines in population longevity.

### Individual vs. Population Approaches to Anxiety, Depression

From the earliest days of psychiatric epidemiology (58–60), the evidence has been robust and clear that lower socioeconomic groups evidence higher rates of mental disorder in a dose-response, relatively linear gradient. This traditional literature has often been interpreted in materialistic terms, but more analytical researchers such as Hollingshead and Redlich (61) and Leighton (62) have focused on psychological stress interpretations of the social class-mental health relationship.

Since the period of the Great Depression, Marie Jahoda, Peter Warr, and other sociologists (63, 64) have focused on the disintegrative social and psychological effects of losses of employment and its meaning in terms of damage to identity, self-esteem, social relations, and social support. Following the epidemiological studies of Hollingshead and Redlich in New Haven, Brenner found that, for over a century and a half, mental hospitalization coincided with decreases in employment in New York State (65). This early macroeconomic study, explicitly looking at national and regional economic changes, gave rise to the work of more localized studies with smaller samples of the effect of economic loss on mental health indicators, especially by Catalano and Dooley (66, 67). The latter researchers particularly concentrated on the potential circular relationship of the effects of prior mental disorder on job loss, potentially leading to subsequent effect of job loss on deteriorating mental health. At the population level this would mean that the mentally ill-compromised would be more vulnerable to potential job losses during recession, and find it more difficult to retrieve employment when economic recovery subsequently emerged. This duality of approach currently seems to be the more consensual frame of reference in psychiatric epidemiology. Nevertheless, an even more current literature has emerged since the Great Recession, focusing on the downsizing of firms, where it appears clear that job reduction heightens mental health problems (28). And even more novel is the observation that downsizing also has damaging mental health effects on those who remain in employment as recession envelops a firm (29).

### Socioeconomic Status Approaches, Especially Income in Relation to Mental Health

In the history of Western epidemiology, socioeconomic status is perhaps the single most consistent explanatory factor in terms of understanding the distribution of mental health problems. Social determinants have always played a major role in the genesis of physical ill-health but increasingly this is being focused on mental illnesses (68). One of the more detailed recent reviews of the literature demonstrating the interaction of low social class in relation to disturbed mental health, on the one hand, and the influence of the recent industrialized country recession and subsequent austerity policies examines the effect of the Great Recession on a large literature covering multiple industrial country societies in relation to mental health outcomes (69). This review covered 11 studies of alcohol abuse and 8 studies of drug abuse and other addictions; in all of these types of mental health outcomes the very great majority showed elevation of poor mental health coping mechanisms in the face of the recession.

Over the years of development of psychiatric epidemiology, income, occupational skill level and educational level have been successfully used to understand the distribution of a wide variety of mental disorders, from those which have a more definitive genetic and physiological basis, such as schizophrenia, bipolar depression and dementia to those with a broader emotional spectrum, including anxiety, depression, and PTSD. The usual inference has been toward a new interpretation involving traumatic life events, including especially health concerns and financial disturbances as well as harm to social relations, especially those involving family and close friends. These findings for psychiatric illnesses had begun to be developed in the 1930s, coinciding with the Great Depression, which is not unlike the greater concern with physiologic illnesses ranging from infection to cardiovascular disease, becoming especially prominent in the 1970s (70). In fact, however, in the British and other European demographic literatures, we can see that the nearly iron-clad findings of the impact of “social class” on mortality have been observed since at least the 1940s. In those older, heavily physiologic, investigations of mortality, especially in the British Registrar General's Reports, the original observation of what is now called the “social gradient” or “health gradient” has been observed (44). There was little controversy among statisticians and demographic historians over the meaning of the social class-mortality gradient, in which higher occupational skill level of workers correlated very closely with decreasing levels of mortality. The clear interpretation seems to be that the physical stresses of work, environmental exposure, exhaustion and other manifestations of a stressful environment were the likely sources of this relationship.

The general understanding, even thought to be commonsense in the present era, is that a lower level of human resources, including nutrition, an egalitarian and stable public health structure, higher levels of education, render the population more highly adaptable to environmental threats. But the place of income has been more widely recognized in the later twentieth and twenty-first centuries. Within predominantly capitalist cultures, money, and income is seen correctly as the source of ability to purchase virtually every sort of physical commodity and knowledge-based output, health care, intellectual advice and political engagement. Thus, where significant environmental stresses, including those of unemployment and a damaged economy were rampant, clearly the importance of income as a means of escaping from the ravages of deprivation were fairly obvious. Much of the epidemiology—especially in the British case—of social class and illness has been responsible for the more robust development of the European welfare states and the force of the intellectual expression of labor unions.

Similar themes to this emerged as well in the psychiatric epidemiology of the 1950s. However, it was not until a decade or two later that psychiatric epidemiology began to focus on the macro-, or national-level significance of economic changes, especially economic development and unemployment. Moving from the individual level analyses to the macro understanding of economic changes on mental health, the beginnings of econometric analyses using techniques originally developed within the field of economics began to be used (71). The macro findings for the importance of economic events at the national level, but inserted into people's individual lives, have been more recently observed in alcohol and drug abuse.

### Unemployment-Based Approach

Beginning in the 1980s, much less visible but significant long-term economic decline has occurred as a result of the shifting of manufacturing away from the industrialized countries and toward Asia—especially China. This is a phenomenon described very thoroughly by Autor in “The China shock” (72). The resulting economic devastation of manufacturing firms, societies and cultures have given rise to what has been described as “deaths of despair” (73) in this work. The emergence of mortality in younger populations related to alcohol, drugs, the opioid crisis and suicide have not been attributed to spectacular events like recessions, but rather the longer-term destruction of manufacturing employment in the industrial Western world and the emergence of the “gig” economy. Nevertheless, it is clear, from authors such as Autor, Krugmann, Rogoff, and others (74–76) that the clear evidence of many varieties of mental health disturbance have arisen out of an “economic shock” which has turned into a long-term trend, from which no immediate end is envisioned.

### Life Events Approaches

Since the 1950s, one of the more sustained approaches in psychiatric epidemiology has been in the construction of scales identifying distinctive key events in the life cycle that tend to represent major changes (77, 78). The theory here is that significant changes in social role or social position, involving family, work role, friendship patterns, adverse health events sufficiently alter the circumstances of adaptation to the individual environment so that they should be considered stressful, i.e., requiring individual effort or resource expenditure in order to minimize circumstances to which the individual has difficulty adapting and thus could permanently change the person's health status. In this perspective even “good” or positive life changes such as marriage or taking on a new job or promotion with greater responsibilities, constitute a challenge to adaptation in that the person must significantly alter his/her pattern of living to cope with the requirements of the changed life circumstances. Considerable research over multiple generations have used the SSRS, and its many alternate versions, to assess the extent of stress in people's lives and thus attempt to predict heightened illness rates, especially mental health disturbances, emanating from a sum of such life alterations (77, 79). Subsequent researchers have tended to focus more exclusively on negative life events, such has major illnesses or economic losses, with somewhat greater success particularly in predicting negative mental health outcomes (80, 81).

### Quantitative Impact of Great Recession on Mental Health

At the macro level, a substantial number of studies have continued to demonstrate the damaging impact of economic disturbances, especially recessions, involving high levels of unemployment in the United States, Europe and other parts of the industrialized world (43, 82–84).

The question now arises as to whether relatively recent disturbances to the national economy have shown the effects of unemployment and income loss on stress-related chronic disease and mental health disturbances. Findings have demonstrated the effects of losses of wealth on mortality (85) and the effects of both GDP losses and higher unemployment in Europe on increased cardiovascular mortality and self-reported health (86, 87). Additionally, the effects of downsizing on disturbed mental health and alcoholism have been found in national European and US studies (88, 89). These studies of downsizing have been more widely reported, with the additionally interesting finding that during downsizing even workers who maintained their employment showed evidence of disturbed mental health. Potentially most telling, in this respect, is that the most carefully designed Scandinavian studies have demonstrated a circular relationship between unemployment and disturbed mental health, in which, persons with lower mental health scores were more prone to recession-based job loss, and the job loss in turn was related to subsequent increases in mental disorders (29). This type of study appears to have put to rest the question of whether the relationship between poor economic status and poor mental health is causally related to the influence of mental health on later inability to find work or job loss, as distinguished from the situation of job loss making mental health problems more likely. The answer now appears to be that both sequences have a causal place in the relationship between job loss and deteriorated mental health.

The review by Brenner referred to earlier (44), also included studies on suicide which focused on the effects of unemployment. The outstanding methodological problem uncovered in the latter review showed that nearly always the metric used to identify the recessional impact consisted of the unemployment rate. Surprisingly, the recessional factor with the greater potential for damaging health, namely income loss through GDP and median income decline, was not to be found among these studies. The problem here is that while unemployment has been the most journalistically popular reference for the effect of recession, that measure clearly affects a minority of the population, generally <10%, whereas losses of income and wealth affect far greater proportions of the population over a longer period in the life cycle.

### Effects of Economic Loss vs. Those of Unemployment on National Suicide Rates

Since our primary goal in this paper has been to ascertain the importance of changes in the economy, particularly recessions, on suicide, two outstanding observations must be noted. First, the prior literature provides robust indications that increased unemployment is a prime national predictor of suicide rates, and this is reproduced herein for industrialized countries in the Great Recession. But it is clear, that unemployment is not the most important economic predictor of national suicide rates, even though, both journalistically and in academic papers, it is the most frequently researched macroeconomic topic in relation to mental health, going back to original observations in the 1970s. This is true even though in analyses in this article, the oldest populations over 70—in the case of females and over 75 for males—do not show a relationship between national employment and suicide rates. The effects of economic loss during recessions must be seen, primarily, in terms of income loss to families over the short- and long term. Of course, such losses coincide, temporally, with recessions, during which unemployment is also high. But it is clear from these analyses that income losses, even among populations that do not lose employment are the more salient predictors of suicide rates for all age groups. In fact, the most powerful effects of income loss on suicide for both sexes are observed over the age of 70 and are outstanding over the age over 75. Therefore, it is necessary to consider the recessional effects on suicide to include a combination of GDP decline and unemployment increases.

### Relation Between Recession and Suicide Is Underestimated

Secondly, it appears clear from these analyses that national levels of diagnostically identified suicide, in national data, probably represent a considerable underestimate of the actual suicide rate. This can be inferred from multiple literatures dealing with the effects of alcohol addiction and abuse, drug addiction and abuse, as well as poisoning-related mortality, accident-related mortality, as well as fire, heat and hot substances-related mortality (see Table 5). The separate literatures on these topics make clear that a sizable proportion of these “other” deaths can be understood to involve initial suicide intent at some stage in the process leading to mortality. Future analyses may benefit from these considerations of the national underestimate of suicide by taking into account a compendium of sources of mortality related to anxiety and depression as intrinsic motivation. A step in this direction has been taken by Case and Deaton in their designation of depression-related mortality as “deaths of despair,” including such mortality as related to liver cirrhosis (90). Indeed, other literatures go even farther in their investigations of the relationship between economic stress, clinical depression, and cardiovascular illness (91). There is indeed evidence that in the case of alcohol-related mortality and alcohol-related cardiovascular mortality, that GDP declines and unemployment increases are significant predictors (92–94).

### Short-Term vs. Sustained Effects

The major question now is how the experience of the Great Recession for suicide can be forecast (be repeated) as a result of COVID-19 pandemic. The first effect that other journalistic accounts and non-statistical academic papers have strongly suggested is that the immediate effects of losses (fear, deaths) have also materialized in the current COVID-19 limited period.

However, the major effect on mental health and suicide of COVID-19 may well be a fundamentally and indirect corollary—namely, the consequent effect of economic losses due to shutdowns worldwide. Whether suicide and related mental health effects will emanate from the current COVID-19 related recession depends greatly on the length of that recession, as well as efforts to ameliorate the economic situation of people and businesses by governments. Most important will be the duration and intensity of the COVID-19 recession, but this will of course depend on the duration and intensity of COVID-19 itself as its potential secondary and subsequent waves induce an international transmission from countries in the developing world influencing, in a circulatory manner, subsequent effects on industrialized countries.

### Economic and Mental Health Policy Implications

The joint mortality outcomes of the COVID-19 pandemic and its corollary impact on the economic recession-induced mental health impact will depend on specific policies undertaken by individual country governments. The United States, for example, has experienced one of the most severe direct COVID-19-related mortality rates, as well as extremely large increases in unemployment rates, will likely suffer long-term economic declines (via weak economic recovery) and substantial permanent losses of jobs, life time income and wealth (95–97). This will be especially the case if an additional series of government support to maintain jobs, lengthen unemployment insurance, and payments and greatly extend business loans are not granted.

However, current economic policy considerations, taking COVID-19 health outcomes into consideration, still does not consider the indirect corollary implications of the COVID-19 recession in terms of major mental health outcomes and chronic disease mortality. Bearing such corollary health outcomes in mind, policy makers would be wise to include the total health gains to an expansive economic policy along the lines put forward by the International Monetary Fund (IMF), the World Bank and OECD (98–100).

Nevertheless, even under relatively generous economic support of individuals and businesses, it is quite likely that there will be permanent job losses, especially in the industrialized countries, as a result of economic restructuring under conditions of reduced demand. We have begun to see this occur in many of the service industries, such as restaurants and bars, tourism, travel, transportation, entertainment and shopping, and even healthcare (101, 102). Under these circumstances, it would be prudent for governments to be especially alert to expansion of mental health services. The evidence is clear that damage to a population's mental health can have further long-term consequences for reduction of national income and productivity.

Clearly, the most urgent policy effect must be to contain, and perhaps, eliminate COVID-19, but secondarily, and in order to facilitate public health measures, government financial sources of relief need simultaneously to be implemented.

### Limitations

The present analysis is an estimation of the potential secondary effects of the economic recession due to the pandemic on mental health. The study is confined to the entire industrialized country populations of the OECD. Therefore, some of the special risks to suicide which are specific to each country and their sub-regions (e.g., seasonality, built and ambient environment, social integration, religion, ethnic cultures) could not be extracted and tested as control variables at this point. Similarly, since this is an epidemiological study based on aggregated, rather than individual, data, one cannot separately identify genetic differences or specific psychiatric syndromes that could represent individual proneness to suicide, especially in interaction with major economic variables such as national income (GDP per capita) or the unemployment rate.

From this point of view, it is only attributes of national populations that could be entered into the predictive models—more commonly known in the economic, epidemiological, and social science literatures as macro-level analyses. This approach is very much within the theme of the classical Durkheimian view that suicide rates are, to a large extent, attributes of societal, rather than individual, characteristics. Finally, this statistical correlative analysis only permits us to make inferences as to risk factors at the national level, that relate to the magnitude and fluctuations of population suicide rates, rather than enabling inferences as to aspects of causal relations attributed to characteristics of individuals.

Perhaps most important, the statistical analysis has shown that several causes of death are highly predictive of officially designated suicide deaths (see Table 5). These include deaths from poisoning, drug overdoses and unintentional accidents. These relations of differently diagnosed mortality, but potentially harboring an insidious mental state of anxiety, depression or anomie, lead to the conclusion that our suicide models underestimate the degree to which national economic loss and unemployment are risk factors to suicide. The ancillary diagnoses, in which suicidal intent could be a major underlying component were not taken into account as potential suicides. Thus, many “true” suicides, that were labeled differently in the ICD code—due to stigma, religion, unfamiliarity with psychiatric basis of mortality diagnosis or “error”—are likely, according to our findings, to have been substantially underreported. Therefore, the impact of these macroeconomic phenomena on officially designated suicide—even taken as a proxy marker of anxiety and depression—do not fully indicate the magnitude of mental distress brought about by recession in the short and long term.

### The Way Forward

The governments and policymakers have a moral and ethical obligation to ensure the physical health and well-being of their populations. While setting in place preventive measures to avoid infections and then subsequent mortality, the focus on economic recovery has to be taken seriously. What is worrying is that 193 countries appear to be fighting the virus and the pandemic in 193 ways as if the virus requires visa permits which can be denied and the walls can stop the virus. A global pandemic requires a global response with a clear inter-linked strategy for health (5, 17, 103) as well as economic solutions. The vulnerable individuals and economies need to be protected in careful well thought-out ways to support under-privileged groups and communities.

## Data Availability Statement

Publicly available datasets were analyzed in this study. These data can be found here:

GDP per capita: World Bank, International Comparison Program database (https://databank.worldbank.org/source/jobs/Series/NY.GDP.PCAP.PP.KD).

Unemployment rate: ILO (https://www.ilo.org/shinyapps/bulkexplorer46/?lang=en&segment=indicator&id=UNE_2EAP_SEX_AGE_RT_A).

IHME. Global Health Data Exchange (http://ghdx.healthdata.org/gbd-results-tool?params=gbd-api-2019-permalink/9e08d79f2a6f48a4d42fc81f0db67eae').

## Author Contributions

MB developed the conceptualization and statistical analysis, and wrote the main findings in relation to the literature. DB wrote portions of the introduction, relation to psychiatry and mental health services, policies focused on underprivileged populations and The Way Forward, and wrote the abstract and edited the full manuscript. All authors contributed to the article and approved the submitted version.

## Conflict of Interest

The authors declare that the research was conducted in the absence of any commercial or financial relationships that could be construed as a potential conflict of interest.

## Footnotes

^1^See discussion of the use of aggregated data in epidemiological analysis in Szklo and Nieto.
