# Erratum: Acceleration of Anxiety, Depression, and Suicide: Secondary Effects of Economic Disruption Related to COVID-19

**DOI:** 10.3389/fpsyt.2021.660659

**Published:** 2021-02-19

**Authors:** Frontiers Production Office

**Affiliations:** Frontiers Media SA, Lausanne, Switzerland

**Keywords:** COVID-19, economy, suicide, depression, national income loss, unemployment, recession, Great Recession

Due to a production error, there was an error in the affiliations. The affiliations for M. Harvey Brenner should read: 1. Johns Hopkins University Bloomberg School of Public Health, Health Policy and Management, Baltimore, MD, United States. 2. University of North Texas Health Science Center, Fort Worth, TX, United States. 3. Medizinische Hochschule Hannover, Hanover, Germany. The affiliation for Dinesh Bhugra should read: 4. King's College London, London, United Kingdom. The publisher apologizes for this mistake.

The original article has been updated.

